# Patients’ perspectives of acceptability of ART, TB and maternal health services in a subdistrict of Johannesburg, South Africa

**DOI:** 10.1186/s12913-018-3625-5

**Published:** 2018-11-07

**Authors:** Blaise Joy Bucyibaruta, John Eyles, Bronwyn Harris, Gaëtan Kabera, Kafayat Oboirien, Benon Ngyende

**Affiliations:** 1 Centre for Health Policy, School of Public Health, University of the Witwatersrand, Private Bag 2, Wits 2050 Johannesburg, South Africa; 2 Department of Statistics, University of South Africa, PO Box 392, Unisa 0003 Pretoria, South Africa; 3 Department of Homoeopathy, Faculty of Health Sciences, University of Johannesburg, 55 Beit St, Johannesburg, 2028 South Africa

**Keywords:** Access, Acceptability index, Patient-provider interaction, Patient-health service interaction, Patient-community interaction

## Abstract

**Background:**

The field of acceptability of health services is emerging and growing in coherence. But there are gaps, including relatively little integration of elements of acceptability. This study attempted to analyse collectively three elements of acceptability namely: patient-provider, patient-service organisation and patient-community interactions.

**Methods:**

Mixed methods were used to analyse secondary data collected as part of the Researching Equity in Access to Health Care (REACH) study of access to tuberculosis (TB) treatment, antiretroviral therapy (ART) and maternal health (MH) services in South Africa’s public health sector.

**Results:**

Provider acceptability was consistently high across all the three tracer services at 97.6% (ART), 96.6% (TB) and 96.4% (MH). Service acceptability was high only for TB tracer (70.1%). Community acceptability was high for both TB (83.6%) and MH (96.8%) tracers.

**Conclusion:**

Through mixed methods, this paper provides a nuanced view of acceptability of health services.

## Background

Access to antiretroviral therapy (ART), tuberculosis (TB) treatment and maternal health (MH) services in developing countries, including South Africa, remains inadequate and inequitable [1–4]. At a global level, HIV, TB and maternal deaths still represent major challenges to public health [4–6]. In 2016, an estimated 10.4 million people contracted TB, including 1 million people living with HIV; and 1.7 million died, with TB contributing to 33% of HIV-related deaths [2]. By the end of 2017, an estimated 36.9 million people were living with HIV and there were almost 1 million HIV-related deaths [5]. In 2015, approximately 830 women, almost all in low-resource contexts, died every day from causes related to pregnancy and childbirth [4]. Most of these deaths were preventable, linked to factors such as unavailable skilled birth attendants, limited information, distance and inadequate facilities [4]. Finding ways to address barriers to healthcare access is at the heart of current global strategies for tackling HIV, TB and MH mortality [4–6].

South Africa has the world’s sixth highest TB epidemic and it is one of seven countries that collectively account for two thirds (64%) of the world’s TB incidence [6]. In 2016, the country’s TB incidence rate was 438,000 and nearly 60% of people living with HIV/AIDS were estimated to be coinfected with TB [6]. South Africa has the largest ART programme in the world with estimated 7,1 million people living with HIV based on epidemiological statistics published by South African National Aids Council (SANAC) in its 2017/18 Annual Performance Plan [7].

In the past decade, South Africa’s maternal mortality rate (MMR) declined from 189.5 per 100,000 live births in 2009 to 132.9 in 2012/13 [8] and to 119 in 2015 [7]. However, this falls short of the millennium development goal (MDG) of 38 per 100,000 [9] and remains “unacceptably high” [10]. Furthermore, the country is now under pressure to reduce the MMR by 70% by 2030 as per the Sustainable Development Goals (SDGs) [11].

The South African government does recognize these issues and has, since the advent of democracy in 1994, attempted to bring quality health care to all citizens, focusing specifically on the primary and district healthcare systems [11, 12]. But how do these matters affect health services access which is seen as the provision of adequate and non-discriminatory health care to individuals regardless of “who” they are and their circumstances (financial, social, cultural, etc.) [13]?

In this study, access to health services is understood as a dynamic composite concept produced by dimensions of affordability (financial access), availability (physical access) and acceptability (cultural and social access) [14]. While a growing interest in evaluation of access to health services has been noted [15–17], the acceptability dimension, which can be defined as a cultural and social degree of fit between the health system and the users (patients or clients), remains poorly conceptualized [18, 19].

Yet, in South Africa, negative interactions with health providers, including being shouted at, provider inattentiveness and insensitivity, or being turned away in the early phase of labour, may result in women finding maternal services unacceptable [20]. More generally, unproductive and negative interactions between health providers and patients represent the main sources of mistrust [21]. In some cases, disrespectful interactions with providers have resulted in patients doubting their treatment efficacy and consequently switching to traditional healers [21] or defaulting from medical care, even if temporarily [22].

Gilson proposes three elements of acceptability, namely patient-provider interaction, patient-health service organisation interaction and patient-community interaction [23]:

- Patient–health provider interaction: the relationship between the patient and health provider [15, 24], which is understood through the expectations and beliefs from one toward another.
- Patient–health service interaction: the experiences lived by a patient when seeking health services and their perceptions about health service organization and delivery [15], including the length of queues, facility cleanliness and opening hours [23].
- Patient-community interaction: the patient is not isolated but lives in a family and in a community with relatives and friends who might positively or negatively influence the patient’s acceptability of health care [23]. This element draws attention to the roles of family, friends and community often not emphasised enough in understanding the acceptability of health services to patients [23, 24].

**Fig. 1:**
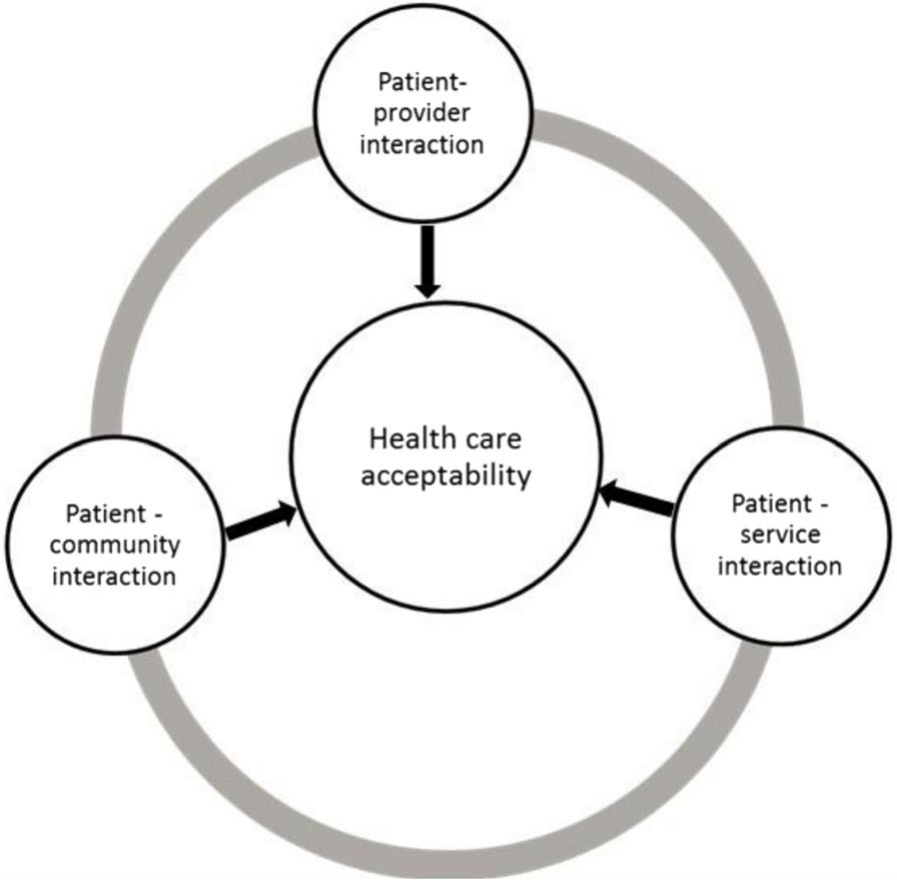
Adapted Conceptual Framework of Acceptability

Although conceptualised separately, these elements are themselves interconnected [23]. Thus, this study aimed at exploring and describing the factors influencing levels of acceptability amongst users of ART, TB and MH services. These services require sustained engagement between healthcare users and providers and thereby provide insight into some of the major health system challenges in the South African context, including how to attract and retain patients within and across services. We thus developed a conceptual framework of acceptability based on existing literature (Fig. 1) [25].

## Methods

### Study design and study population

This study applies mixed methods to secondary quantitative and qualitative data collected as part of the Researching Equity in Access to Health Care (REACH) project, a multi-site, five year study in four of South Africa’s provinces. In this study, we draw on a sub-set of the REACH study population comprised of patients attending HIV, TB and Maternal Health Services from a sub-district in the City of Johannesburg. These data were collected between July 2008 and December 2010, a time of policy change directed towards expanding access to ART, with changes to clinical guidelines and the beginning of service roll-out from specialised community centres to primary health care clinics [26]. Since the study period, ART policy has become increasingly inclusive, culminating most recently in the adoption of WHO Universal Test and Treat Policy, which prescribes treatment on diagnosis, regardless of clinical indicators [27]. Therefore, while we recognize that this dataset may seem dated, given the sheer scale of South Africa’s ART programme and inclusive treatment policy environment [11, 27], we assert its continued importance for practice, policy and methodological development.

### Data description

The quantitative data consisted of patient exit interviews including socio-economic and demographic background, dwelling characteristics, household income, expenditure, household assets and acceptability of care. Furthermore patients’ self-reporting of their clinical conditions were considered: for ART services: buddy or support group, frequency of treatment collection, frequency of forgetting/not taking ART; for TB treatment: Directly Observed Treatment Short-course (DOTS) checked, frequency of TB medication collection, forgetting collecting/drinking TB medication and missing visit; and for MH: maternal parity, HIV status and type of delivery.

The qualitative data consisted of in-depth interviews covering the participant’s illness (HIV and TB)/pregnancy and access stories, including exploration of the acceptability of care expected and received (MH).

### Data management

With regard to quantitative data, the three main acceptability constructs were developed, namely patient-provider interaction, patient-health service organization interaction and patient-community interaction. The acceptability variables were recoded in order to pool and categorise them on a binary scale with values coded “1” for a positive response and “0” for a negative response.

With respect to qualitative data, in recognition that the researchers’ experience, background and expectations would affect, at least to some degree, the interpretation of the narratives from the in-depth interviews [28], we collectively developed a thematic coding system to ensure coding agreement. This coding system took into consideration the context in which those in-depth interviews took place. The acceptability themes included “perceived patient-health provider interaction”, “perceived patient-health care organization interaction” and “perceived patient-community support”.

### Statistical analysis

Quantitative data analysis was conducted using STATA version 14. Unit weighted composite scores were used to develop acceptability indices. A composite score was calculated as the average of coded responses in each of the three acceptability constructs. This method is regarded as an adequate method for developing a composite index [29, 30]. For ease of interpretation, the composite index was multiplied by 100 so that each composite index was expressed as percentages. The acceptability index was computed by dichotomising the composite indices as follows: the low acceptability index was defined as ranging from 0 to 66.66%, while the high acceptability index was above 66.66%. This cut off was also guided by acknowledging the patients’ fear to give a negative opinion about health provider or health services [31, 32].

Binary simple logistic regression was used to determine factors associated with the acceptability index. Then, all factors with a *p*-value less or equal to 0.20 in the univariate analysis were included in adjusted multiple logistic regression model. A *p* value < 0.05 was considered to indicate statistical significance.

Regarding qualitative data, in-depth interview transcripts were imported into MAXQDA.12 to assist thematic content analysis. The narratives were reviewed and analysed deductively using the ‘acceptability themes’ from the conceptual framework (Fig. 1) and related to the ‘acceptability constructs’ used in quantitative analysis. Simultaneously, inductive analysis was done to consider the new themes emerging from the transcripts.

To allow a deeper understanding of the results from this mixed study analysis, triangulation was used to integrate the findings from both quantitative and qualitative methods during the discussion of the results. Triangulation is a method that facilitates the verification of data through cross validation from more than two sources, and is recommended for mixed study analysis [33].

## Results

This study analysed quantitatively 987 patients’ exit interviews: 331 for ART, 297 for TB and 359 for MH services. Qualitative analysis consisted of 15 in-depth interviews, 8 for ART/TB and 7 for MH services population.

### ART: Survey

Table 1 summarises the quantitative results of ART Tracer acceptability.

**Table 1:** Key quantitative results for ART Tracer

Variables	Provider acceptability				Service acceptability				Community acceptability			
	Simple (unadjusted) logistic regression		Multiple (adjusted) logistic regression		Simple (unadjusted) logistic regression		Multiple (adjusted) logistic regression		Simple (unadjusted) logistic regression		Multiple (adjusted) logistic regression	
	OR (95%CI)	*P*	OR (95%CI)	*p*	OR (95%CI)	*p*	OR (95%CI)	*p*	OR (95%CI)	*p*	OR (95%CI)	*p*
Age group												
≤ 40	Ref											
≥ 41	3.1 (0.37–26.45)	0.292	–	–	1.7 (1.07–2.86)	0.026	1.5 (0.79–2.96)	0.211	0.7 (0.38–1.39)	0.342	–	–
Gender												
Female	Ref											
Male	0.3 (0.10–2.81	0.457	–	–	1.9 (1.09–3.45)	0.023	2.6 (1.18–5.73)	0.018	1.1 (0.49–2.49)	0.814	–	–
Education												
No schooling	Ref											
Primary	8.4 (0.69–102.08)	0.095	25.0 (0.41–1518.62)	0.124	1.4 (0.42–4.69)	0.586	–	–	0.6 (0.15–1.53)	0.111	–	–
Secondary	11.6 (1.90–71.29)	0.008	11.8 (0.84–166.49)	0.068	0.8 (0.26–2.39)	0.675	–	0.4 (0.37–2.83)	0.308	–	–	
Tertiary	–	–	–	–	–	–	–	–	0.3 (0.12–4.91)	0.398	–	–
Employment												
Employed	Ref											
Unemployed	7.3 (1.38–38.24)	0.019	18.3 (1.65–202.05)	0.018	1.4 (0.26–2.43)	0.226	–	–	0.6 (0.32–1.23)	0.173	0.5 (0.23–1.180)	0.121
SES												
Low	Ref											
Middle	15.2 (1.53–151.36)	0.020	38.9 (2.01–754.32)	0.015	1.5 (0.63–3.49)	0.363	–	–	0.9 (0.38–2.52)	0.971	–	–
High	3.9 (0.75–20.49)	0.104	8.3 (0.65–103.98)	0.102	1.7 (0.71–4.03)	0.238	–	–	1.9 (0.71–4.98)	0.202	–	–
Home location												
Far	Ref											
Near	–	–	–	–	0.4 (0.24–0.69)	< 0.001	0.3 (0.12–0.56)	0.001	0.6 (0.29–1.39)	0.258	–	–
Means of transport												
Walking (foot)	Ref											
Public transport	9.4 (1.78–49.77)	0.008	5.5 (0.69–44.67)	0.105	0.6 (0.34–0.99)	0.049	0.8 (0.36–1.78)	0.592	1.2 (0.61–2.50)	0.552	–	–
Missed ART doses												
No	Ref											
Yes	0.7 (0.15–4.09)	0.766	–	–	0.5 (0.28–0.99)	0.049	0.6 (0.27–1.37)	0.234	0.9 (0.46–2.02)	0.928	–	–
ART support group												
No	Ref											
Yes	0.4 (0.93–1.99)	0.280	–	–	0.6 (0.29–1.14)	0.111	0.8 (0.38–1.77)	0.608	2.4 (1.07–5.34)	0.033	2.5 (1.10–5.63)	0.029

#### Provider acceptability

Simple logistic regression analysis showed that the odds of ART-Provider acceptability was higher for individuals with secondary school than those without schooling (OR = 11.6), for unemployed people compared to their employed counterparts (OR = 7.3) and those from middle SES compared to those from a low SES (OR = 15.2). It was further noted that the individuals who used public transport had higher odds for ART-Provider acceptability than those who walked to health facility (OR = 9.4).

Multiple logistic regression analysis showed that the odds of ART-Provider acceptability was higher for unemployed compared to employed people (OR = 18.3). It was also noted that the individuals from a middle SES had higher odds for ART-Provider acceptability than those from a low SES (OR = 38.9). Moreover, the odds of ART-Provider acceptability was higher for patients attending the primary health facility than those attending tertiary hospital (OR = 6.9).

#### Service acceptability

Simple logistic regression analysis showed the patients aged 41 years and above had higher odds for ART-Service acceptability than those aged 40 years and below (OR = 1.7), for male patients compared to females (OR = 1.9) and for those attending at Primary Health Care (PHC) compared to those attending at tertiary hospital (OR = 3.2). However, the odds of ART-Service acceptability was lower for patients whose home was located near to health facility than those whose home was located far from the health facility (OR = 0.4), those who used public transport compared with those who walked to health facility (OR = 0.6), and for those who missed ART-dose against those who did not OR = 0.5).

Multiple logistic regression analysis showed that male patients (OR = 2.6), patients attending the primary health facility than those attending tertiary hospital (OR = 5.7) compared to their counterparts. However, the odds of ART-Service acceptability was lower for patients whose home location was near than those whose home location was far from the clinic (OR = 0.4).

#### Community acceptability

Simple logistic regression analysis showed ART support group as the only factor associated with ART-Community acceptability (OR = 2.4). It remained statistically significantly in multiple logistic regression analysis (OR = 2.5).

### TB treatment: Survey

Table 2 presents the summary of TB Tracer acceptability results.

**Table 2:** Key quantitative results for TB Tracer

Variables	Provider acceptability				Service acceptability				Community acceptability			
	Simple (unadjusted) logistic regression		Multiple (adjusted) logistic regression		Simple (unadjusted) logistic regression		Multiple (adjusted) logistic regression		Simple (unadjusted) logistic regression		Multiple (adjusted) logistic regression	
	OR (95%CI)	*p*	OR (95%CI)	*p*	OR (95%CI)	*p*	OR (95%CI)	*p*	OR (95%CI)	*p*	OR (95%CI)	*p*
Age group												
≤ 40	Ref											
≥ 41	1.8 (0.36-8.81)	0.473	–	–	1.0 (0.62-1.78)	0.865	–	–	0.5 (0.19-1.06)	0.068	0.6 (0.23-1.37)	0.206
Gender												
Female	Ref											
Male	0.9 (0.23-3.32)	0.840	–	–	1.5 (0.92-2.52)	0.099	1.8 (0.97-3.26)	0.064	0.3 (0.13-0.88)	0.027	0.4 (0.14-1.09)	0.074
Education												
no schooling	Ref											
Primary	–	–	–	–	0.4 (0.05-4.02)	0.473	–	–	0.4 (0.04-4.35)	0.471	–	–
secondary	–	–	–	–	0.4 (0.04- 3.06)	0.351	–	–	1.8 (0.19-17.19)	0.610	–	–
tertiary	–	–	–	–	–	–	–	–	–	–	–	–
Employment												
Employed	Ref											
Unemployed	1.7 (0.41-6.91)	0.474	–	–	1.3 (0.74-2.29)	0.354	–	–	0.7 (0.24-1.74)	0.390	–	–
SES												
Low	Ref											
Middle	–	–	2.1 (1.43-7.91)	< 0.001	0.4 (0.12-1.11)	0.075	0.4 (0.12-1.43)	0.165	0.3 (0.04-2.66)	0.294	–	–
High	2.5 (1.56-4.57)	< 0.001	–	–	0.7 (0.23-2.32)	0.588	1.1 (0.29-3.89)	0.910	0.6 (0.06-5.02)	0.615	–	–
Home location												
Far	Ref											
Near	–	–	–	–	0.5 (0.11-2.41)	0.394	–	–	3.4 (0.75-14.94)	0.115	3.8 (0.74-18.90)	0.109
Means of transport												
Public transport	Ref											
Walk (foot)	0.6 (0.08-5.10)	0.658	–	–	0.6 (0.23-1.19)	0.136	0.4 (0.18-1.89)	0.910	0.8 (0.23-3.06)	0.784	–	–
Pre-treatment smear results												
Negative	Ref											
Positive	3.3 (0.60-16.87)	0.172	3.2 (0.58-17.07)	0.181	0.7 (0.39-1.27)	0.246	–	–	1.0 (0.41-2.49)	0.982	–	–
Pre-treatment culture results												
Negative	Ref											
Positive	2.3 (0.13-38.12)	0.571	–	–	1.2 (0.26-5.12)	0.847	–	–	2.0 (0.52-7.99)	0.308	–	–
DOT checked												
No	Ref											
Yes	–	–	–	–	0.2 (0.13-0.42)	< 0.001	0.2 (0.09-0.34)	< 0.001	1.7 (0.72-3.92)	0.234	–	–
Missing visit^a^												
No	Ref											
Yes	–	–	–	–	2.2 (1.09-4.31)	0.027	2.1 (0.94-4.71)	0.071	2.8 (0.61-12.53)	0.186	2.5 (0.51-12.68)	0.256
Missing visit^b^												
No	Ref											
Yes	–	–	–	–	4.7 (1.39-15.95)	0.012	5.1 (1.40-18.67)	0.013	1.1 (0.22-5.21)	0.932	–	–

visit^a^: visits during intensive phase of the TB treatment (first 2 months); visit^b^: visits during continuation phase of the TB treatment (after the first 2 months)

#### Provider acceptability

Except SES, both simple and multiple logistic regression analysis did not show any factor statistically associated with TB-Provider acceptability.

#### Service acceptability

Simple logistic regression analysis showed that individuals whose DOTS was checked had lower odds (OR = 0.2) while those who missed their clinical visits during intensive (first two months) (OR = 2.2) and continuous (after the first 2 months) (OR = 4.7) phase of their treatment had higher odds of service acceptability than their comparator groups. Similar findings were found in multiple regressions.

#### Community acceptability

Simple logistic regression analysis showed that male patients had lower odds (OR = 0.4) for TB-Community acceptability compared to females. Multiple logistic regression models added no other significant relationships.

### Perceptions and experiences of ART and TB services: In-depth interviews

The qualitative findings enrich these pictures but ART and TB patients were not separated for in-depth interviews. Eight patients aged from 23 to 51 years, took part in in-depth interviews to explore their acceptability of ART and/ or TB health services.

#### Perceived patient-provider interaction

Only two of the eight patients explained that particular health workers (HWs) had been kind and nice to them. The remaining six patients perceived nurses as rude and having negative attitudes. Two saw this as leading to defaulting from their treatment.

*…I was not ok with that then after two or three days I came back here and told her how I feel about what she did. And she only shouted back asking me why am I there at that time to collect the pills and that’s when I decided to give up the pills* (U2-V-ART-TB-PA1).

While unacceptable provider-patient interactions contributed to these cases of defaulting, there were other reasons why patients stopped their treatment (even if temporarily), namely side effects of the medication (U2-BV-TB-ART-PA8), feeling better (U2-V-ART-TB-PA1), job commitments, especially working night-shift (U2-V-ART-TB-PA1) and health system issues (U2-V-ART-PA5).

#### Perceived patient-service interaction

Participants noted that space and infrastructure did not always enable an acceptable service. For example, in one clinic, infrastructural constraints forced the counsellors to provide confidential information such as HIV status while other patients were present.

*…The clinic is always full. There are children’s clinic here and there is family planning clinic and there are those people who are here to get their treatment too so they were mixed. When high blood people* [i.e. with hypertension] *were still coming they used to sit on that side and there would not be any space here because there were many people* (U2-B-TB-ART-PA7).

Queues were generally seen as being long. While waiting in lines, patients had opportunity to talk about their illness but there was a feeling that multiple and inappropriate queues reflected a general lack of patient centredness.

*…The conversation that we are talking about is HIV nothing else and the way that the sisters* [i.e. nurses] *are not treating people in the right way. The question of not treating people in the right way was due to the way people were seating. We sit in the wrong queues* (U2-D-ART-PA4).

#### Perceived patient- community interaction

Only two patients said that they had family and/or friend support for their illness and treatment needs, and the rest did not.

While most of the patients had disclosed their HIV status to a family member or friends (e.g. U2-V-ART-TB-PA3 and U2-D-ART-PA4), several felt stigma and judgment from the wider community toward HIV positive patients

*…Criticism was the only reason why I was afraid for people to see me and telling me that I have AIDS... what mostly concerned me was my career, my friends and family, my girlfriend almost everyone that was in my life because I thought they would reject me or keep me away from them because I was sick* (U2-B-ART-TB-PA6).

### Maternal health services: Survey

Table 3 presents a summary of quantitative results of MH Tracer acceptability.

**Table 3:** Key quantitative results for MH Tracer

Variables	Provider acceptability				Service acceptability				Community acceptability			
	Simple (unadjusted) logistic regression		Multiple (adjusted) logistic regression		Simple (unadjusted) logistic regression		Multiple (adjusted) logistic regression		Simple (unadjusted) logistic regression		Multiple (adjusted) logistic regression	
	OR (95% CI)	*p*	OR (95% CI)	*p*	OR (95% CI)	*p*	OR (95% CI)	*p*	OR (95% CI)	*p*	OR (95% CI)	*p*
Age group												
≤ 20	Ref											
21–30	0.6 (0.08–5.05)	0.655	–	–	3.8 (1.39–10.11)	0.009	2.4 (0.86–6.72)	0.094	1.3 (0.15–12.40)	0.792	–	–
≥ 31	1.1 (0.10–12.97)	0.917	–	–	3.4 (1.17–9.65)	0.024	1.5 (0.47–4.95)	0.486	0.4 (0.05–3.55)	0.411	–	–
Parity												
Nullipare	Ref											
Primipare	1.2 (0.30–4.41)	0.834	–	–	1.7 (0.99–3.03)	0.052	1.7 (0.95–3.13)	0.074	0.2 (0.02–1.42)	0.101	0.2 (0.02–1.68)	0.135
Multipare	1.3 (0.31–5.75)	0.694	–	–	2.3 (1.27–4.05)	0.005	2.3 (1.09–4.62)	0.028	0.2 (0.02–1.59)	0.122	0.2 (0.02–1.85)	0.153
Education												
Primary	Ref											
Secondary	–	–	–	–	–	–	–	–				
Tertiary	0.5 (0.56–3.91)	0.484	–	–	2.7 (0.99–7.21)	0.053	2.6 (0.92–7.29)	0.071	0.5 (0.06–3.97)	0.490	–	–
Employment												
Unemployed	Ref											
Employed	0.4 (0.12–1.21)	0.101	0.4 (0.11–1.33)	0.134	1.0 (0.63–1.65)	0.933	–	–	0.9 (0.24–3.16)	0.834	–	–
SES												
Low	Ref											
Middle	7.5 (1.16–48.51)	0.034	8.0 (1.11–58.41)	0.039	1.5 (0.51–4.52)	0.451	–	–	1.3 (0.15–11.43)	0.800	–	–
High	2.5 (0.48–13.32)	0.274	2.8 (0.46–16.57)	0.267	1.2 (0.40–3.65)	0.731	–	–	4.3 (0.37–49.89)	0.246	–	–
Facility												
Tertiary hospital	Ref											
Primary health centre	1.7 (0.21–13.29)	0.629	–	–	3.8 (1.56–9.17)	0.003	2.7 (1.08–6.96)	0.033	1.2 (0.15–10.22)	0.841	–	–
Home location												
Far	Ref											
Near	1.3 (0.42–4.31)	0.624	–	–	1.3 (0.85–2.12)	0.210	–	–	0.7 (0.18–2.41)	0.536	–	–
Means of transport												
No ambulance	Ref											
Ambulance	0.5 (0.17–1.73)	0.298	–	–	0.9 (0.55–1.38)	0.550	–	–	2.9 (0.59–13.71)	0.188	2.2 (0.042–11.30)	0.358
Attended ANC												
No	Ref											
Yes	–	–	–	–	0.5 (0.14–1.53)	0.204	–	–	8.1 (1.51–43.91)	0.015	1.3 (0.09–17.11)	0.838
Told pregnancy warning signs												
No	Ref											
Yes	–	–	–	–	0.9 (0.33–2.65)	0.901	–	–	8.2 (1.93–34.91)	0.004	7.3 (0.86–62.83)	0.069
Perceived well-managed delivery												
No	Ref											
Yes	7.7 (2.29–25.99)	< 0.001	7.9 (2.24–28.08)	< 0.001	1.1 (0.52–2.28)	0.818	–	–	0.9 (0.11–7.10)	0.899	–	–

#### Provider acceptability

Simple logistic regression analysis showed that the odds for this acceptability was higher for the mothers from a middle SES (OR = 7.5) and those who perceived that their pregnancy was well-managed (OR = 7.7) compared to their counterparts. Similar results can be noted in multiple regressions.

#### Service acceptability

Simple logistic regression analysis showed that older mothers as well as multiparous ones were more likely to rate service acceptability higher. So too did those attending a clinic as opposed to a hospital (OR = 3.8). Similar, but less powerful, associations were found in multiple regressions.

#### Community acceptability

Simple logistic regression analysis showed that community acceptability was about eight times higher for those who attended ANCs and who were educated about pregnancy. Issues. Multiple logistic regression analysis failed to show any factor statistically associated with MH-Community acceptability.

#### Perceptions and experiences of maternal health services: In-depth interviews

Qualitative findings for MH tracer are based on interviews with seven mothers, aged from 20 to 32 years,

#### Perceived mother-provider interaction

Patient-health provider interactions were generally perceived as a barrier to acceptable maternal health care services. Three out of seven mothers expressed their fear to attend antenatal services because of rumours that nurses would be very rude, insensitive (U2-A-CEOC-PA10) or judgemental:

*…I should go to the clinic you see, but then I thought that they were going to shout at me and accuse me of not booking in time and maybe not even attend me, so I told myself that I will see what I do if it takes for me to give birth by myself then so be it because it doesn’t seem like I have a choice* (U2-A-CEOC-PA9).

Many spoke of witnessing or directly experiencing disrespectful interactions with healthcare providers:

*… And even the way they were treating us it was not a proper way of treating other people. We don’t deny that we are there to get help but that is not the way they should offer us their help… they don’t respect us at all, it’s like we are there to bother them, you can’t wait to be out of the clinic sometimes because of the way they treat people* (U2-A-CEOC-PA13).

Feeling powerless in the face of health providers’ negative attitudes and actions, came out many times in the mothers’ narratives. For example, U2-A-CEOC-PA13 said that pregnant mothers are used to nurses’ rudeness and other bad care and must acquiesce because they cannot do anything about it. U2-A-CEOC-PA12 thought those pregnant mothers could not complain or take legal action, because this may affect their future care.

#### Perceived mother-service interaction

Five out of seven mothers perceived that the health service was inadequately organized. In some instances there were drug stock outs and three mothers said that this led to no pain relief (U2-A-CEOC-PA12). Furthermore one mother had to buy a pregnancy test kit from a private pharmacy as none were available at the public clinic.

Emergency services were generally perceived negatively. In some cases ambulances came after a long waiting time or simply did not come at all, especially during the night or into certain areas such as hostels which were perceived to be dangerous.

*… But the ambulance did not come till the next day … you can die while waiting for an ambulance to come get you* (U2-A-CEOC-PA12).

#### Perceived mother-community interaction

Four women confirmed they got strong support from the father of their children and support from their families:

*… I have a boyfriend who was very supportive…with my boyfriend support I started getting over that anxiety and worry about the implications of* [HIV] *positive and pregnant… At home, it* [pregnancy] *did not affect my family life as much, instead It brought them together… all of sudden my younger brother who could not* [usually do anything for me] *without complaining…, but when I was pregnant I asked for a glass of water he would jump! ... I had a very strong support system, without my close family and my boyfriend I don’t honestly think that I would have survived* (U2-A-CEOC-PA10).

The remaining three mothers reported lack of support (financial and emotional), or even conflict with their families or partners.

An emergent theme from the narratives was that pregnancy is not separate from daily life. Often a pregnant woman is expected to look after the house and do all the chores such as cleaning, laundry and cooking, as well as care for household members, even while heavily pregnant. This situation can be very stressing as U2-A-CEOC-PA13 said:

*… Then my sister started troubling me I think I got stressed a month before last, I was much stressed even this month especially this month and I think that is why I gave birth before time. I got stressed and couldn’t cope because she was really sick.*

## Discussion

Drawing on the findings, overall acceptability was generally low among the ART population compared to higher levels of overall acceptability noted among TB and MH tracer population. These results are in agreement with the findings from a study conducted in KwaZulu-Natal as part of the broader REACH project that reported low acceptability of care among HIV positive patients [34]. This low acceptability among the ART population is partly explained by its chronic nature as well as significant stigma in the early 2000s. While HIV services have since become more widely available, and there are more trained staff, recent studies show the remaining and increasingly broad-based impacts of HIV stigma [35, 36]. HIV-related stigma remains a social and health system concern [37] and the roll-out of services with no additional health human resources may also increase provider strain [38].In contrast, stigma seemed less of an issue for those affected by TB – a finding borne out in Roger’s study, carried out at the same time, on stigma among co-infected ART and TB patients in South Africa [39]. This, together with the ‘short-term’ nature of the treatment and curability of TB, alongside greater service availability at primary healthcare level, may explain the relatively higher overall acceptability of TB-services. However, post-REACH, given the proximity of TB to HIV and the emergence of multi-drug resistant TB, there is growing evidence for TB-related stigma, including amongst healthcare workers themselves [40]. Our findings therefore need contextualisation and their relevance understood in relation to the nuances of acceptability as a dynamic concept, closely intertwined with issues of vulnerability, identity and social exclusion [36].

With reference to levels of MH-overall acceptability expressed in the quantitative findings, despite a few stillbirths, most of the mothers noted high acceptability – not unexpected given that a successful delivery is often perceived as a positive outcome by patients and communities. Additionally, mothers’ perceptions of childbirth and perinatal care are more likely to be ‘good’ when they are supported by their family and / or community [41].

### Acceptability of what?

Our findings allow us to address different types of acceptability, namely provider, service and community. With regard to provider acceptability, there is high acceptability across the three services. This contrasts other studies reporting unacceptable patient-provider interactions [34, 42, 43]. But some of these attribute poor provider acceptability to only one aspect of provision such as provider disrespect toward the patient [44] or provider dislike or inattention toward the patient [42]. However in our study, an attempt was made to consider a range of interrelated aspects of provider acceptability (respect, privacy, confidence, being shouted or hit, etc.).

Service acceptability was higher for TB than both ART and MH. For ART, low acceptability may be explained by long, often confusing, queues and the centralisation of the service at the time of the study (offered at specialised community health centres). In contrast, the ‘convenience’ of available TB services – offered in primary health care clinics – may explain their higher acceptability. Chimbindi and colleagues similarly found higher satisfaction for TB services than ART services in the REACH data from KwaZulu Natal Province, with 65% of HIV patients reporting the long queues to see the health worker compared to only 40% of TB patients [34]. Although antenatal services were as available as TB services through primary health care clinics, complex pregnancies and delivery services were (then, as now) only offered at specialised midwife obstetric units and hospitals. Thus low service acceptability may be partially explained by the poor quality of ambulance services. A lengthy distance to facilities is a recognised barrier to maternal health in Africa where transport during childbirth –even in a well-resourced urban area (as in the study site) - remains a challenge [45–47]. Shortages of beds, staff and equipment for maternal health services are also well-documented problems in developing countries [48], including South Africa [46, 49].

### Acceptable to whom?

The association between acceptability and several individual characteristics such as age, gender, marital status, education level and economic status was assessed. Age was an important factor associated with high acceptability of health services for ART and MH tracers. For ART, patients over 40 years had higher odds for MH-Service acceptability than those aged 40 years and younger in unadjusted regression model. These results are consistent with a systematic review which revealed that the older age category was associated with high acceptance of starting ART in sub-Sahara Africa [50]. Yet, Schatz and Knight caution that older patients (above 50 years) may only present to ART services on referral, when they are symptomatic, or when a partner is diagnosed and that this has implications for developing age-appropriate (and thereby, acceptable) HIV testing messaging, with more research needed for reaching this group [51].

Compared to mothers aged 20 years and younger, the odds of MH-Service acceptability were higher for those aged 21 and above. In their recent systematic review, Yakubu and Salisu identify “non-friendly adolescent reproductive services” as a negative health service-related determinant of adolescent health during pregnancy in sub-Saharan Africa [52]. In South Africa, Fatti and colleagues found that adolescent mothers were more likely to present to the health service for the first time during labour, without attending antenatal care [53]. The same study also revealed that young women had reduced rate of antenatal ART uptake -reflecting poor uptake of maternal health services (which led South Africa’s roll out of ART services) [53]. Low levels of acceptability for MH-Service among teen or young mothers may further relate to higher levels of healthcare provider stigma expressed towards younger women for unwanted pregnancies [54], alongside ‘unfriendly’ services for this age group overall [52]. Age is significant due in part to the longer exposures to and experiences of services that aging brings [55], as well as the ways in which healthcare providers perceive and engage with patients of different ages.

Gender was also important in acceptability. Men found ART services more acceptable than women did. This is in contrast to systematic review findings from Mugglin and colleagues that male patients in sub-Saharan Africa were less satisfied with ART services and less likely to be retained in care than women [50]. Zachariah and colleagues similarly found considerable attrition in the ART preparation phase among male patients in Malawi and Kenya [56]. Furthermore, Magnus and colleagues reported that women are more likely to have high HIV stigma score compared to men [42]. This situation could hamper HIV-service acceptability for female patients. Studies have emphasised gender inequities as a key driver of HIV/AIDS, with women bearing the brunt of the epidemic [57]. Gender was also associated with acceptability for the TB tracer. Similar to findings from another REACH-based study conducted in KwaZulu Natal sub-district [34], we found that male patients had lower odds for TB-Community acceptability than females. These results suggest the need for ongoing research into the gendered manifestations of acceptability in health systems and complex patriarchal communities, such as those in South Africa in which men often dominate and women suffer [58].

Socio-economic status (SES) showed considerable influence on patients’ acceptability of health care provider. The current study found that individuals from a middle SES had higher odds for ART-Provider acceptability than those from a low SES for whom health care access means proportionately higher costs. A meta-analysis of sub-Sahara Africa studies found that patients with lower SES were less likely to start ART and had a higher rate of loss to ART program [50].The results also revealed that the odds of TB-Provider acceptability was higher for patients from a middle SES than their counterparts from a low SES. Furthermore, it was noted mothers from a middle SES had higher odds for MH-Provider acceptability than their counterparts from a low SES. These results are consistent overall growing socio-economic inequities in access to maternal health care in South Africa [59].

## Conclusion

The field of acceptability of health services is emerging and growing in coherence. But there are gaps, including relatively little integration of elements of acceptability. Most previous studies had focussed on particular elements of acceptability in isolation. Guided conceptually and methodologically by an acceptability framework, the current study attempted to analyse collectively three elements of acceptability namely: patient-provider, patient-service organisation and patient-community interactions. We focussed on TB, ART and MH services, as complex, closely intertwined conditions requiring sustained engagement with the health system. With the growing global burden of non-communicable diseases (NCD), an application of this methodological approach to the acceptability of NCD services would be highly relevant and timely [60].

We assert that this interrelated approach to acceptability provides not only methodological novelty but also can be used as an important policy tool.

To enhance patient-provide interactions that inspire trust, confidence and empathy - utmost values of health professionals - healthcare providers and policymakers must be patient-centred. But frontline relationships are only one element of acceptability. We recognize that there exist systemic constraints of limited training, shortages of staff and dysfunctional organizations in clinics and hospitals which limit provider capacities even when they try to implement patient-centred care [61].

For health policymakers and health system managers, an ‘acceptability lens’ may help in implementing and monitoring a number of existing health policies –including the Patients’ Rights Charter, Comprehensive Primary Healthcare Service, District Hospital Service Package, National Core Standards for Health Establishments and the National Health Insurance Essential Health Package [11, 62–65]. Some of these policies mention patient involvement but most do not. If ‘acceptable care’ is only paid lip service at the policy level, what can we expect of practitioners and providers? Besides the limitations due to secondary data analysis, the sample sizes were small in some groups as consequence the CI of odds rations were too wide. Therefore, further studies using primary data on larger sample sizes are recommended. 

## Abbreviations

AIDS: Acquired Immunodeficiency Syndrome
ANC: Antenatal Clinic
ART: Antiretroviral Therapy
CI: Confidence Interval
DOTS: Directly Observed Treatment Short-course
HIV: Human Immunodeficiency Virus
HIV/AIDS: Human Immunodeficiency Virus Infection and Acquired Immune Deficiency Syndrome
HWs: Health Workers
MDG: Millennium Development Goal
MH: Maternal Health
MMR: Maternal Mortality Rate
NCD: Non-Communicable Diseases
No: Number
OR: Odd Ratio
PHC: Primary Health Care
REACH: Researching Equity in Access to Health Care
Ref: Reference
SANAC: South African National Aids Council
SDGs: Sustainable Development Goals
SES: Socio-Economic Status
TB: Tuberculosis
UNAIDS: The Joint United Nations Programme on HIV/AIDS
UNDP: United Nations Development Programme
WHO: World Health Organization
